# Nutritional strategies for bone stress injury prevention in military trainees: historical evolution, current evidence, and future directions

**DOI:** 10.3389/fnut.2026.1927763

**Published:** 2026-10-14

**Authors:** Nicole M. Sekel, Stefan M. Pasiakos

**Affiliations:** Pennington Biomedical Research Center, Louisiana State University, Baton Rouge, LA, United States

**Keywords:** injury, micronutrient, military, musculoskeletal, nutrition, prevention

## Abstract

Bone stress injuries exist along a continuum from stress reactions to stress fractures. They carry prolonged recovery times, high recurrence risk, and substantial economic burden, and they pose a direct threat to military readiness. Nutritional status modifies injury susceptibility by influencing skeletal adaptation to mechanical loading, bone modeling and remodeling, and structural integrity. Yet the historical evolution and effectiveness of nutritional policies intended to reduce the bone stress injury prevalence in the military remain poorly understood. In this narrative review, we critically evaluate that evidence base and subsequently propose novel strategies to mitigate injury risk and promote skeletal resilience in military personnel.

## Introduction

Bone stress injuries result from the inability of normal bone to withstand repetitive, submaximal loading (1). They remain the most common overuse injury leading to medical discharge of military trainees (2–4) and impose substantial economic burden across services (5–7), despite extensive injury mitigation efforts over the past several decades (5, 8, 9). Although bone stress injuries were first described over a century ago (10), their pathophysiology continues to be elucidated. The prevailing theory holds that fatigue damage generated by abrupt increases in training volume, such as when a new trainee begins their military service, elicits a temporally and spatially coordinated remodeling response that repairs tissue damage while paradoxically weakening bone (11, 12). In this state, sustained mechanical loading without sufficient rest can perpetuate a cyclical injury process in which accumulated microdamage necessitates further remodeling, prolonging recovery time and increasing susceptibility to reinjury. Diagnoses of bone stress injuries are also often uncertain (13), and early prospective work showed that a substantial proportion of trainees were asymptomatic at the time of diagnosis, complicating prevention and early detection strategies (14). Research examining the etiology of bone stress injuries has identified both non-modifiable factors, such as sex, race, age, and genetics, and modifiable factors, most notably sleep hygiene, training load, timing and preparation, energy availability, and nutritional status (15–17).

Several descriptive epidemiologic studies indicate that bone stress injury may be related to nutritional deficiencies (18–21). However, nutrition-focused bone stress injury prevention strategies in the military have historically relied on a narrow set of isolated micronutrients, namely calcium and vitamin D, which together represent one of the most extensively studied nutritional interventions for bone stress injury prevention in military (22–25), athletic (26–28), and aging populations (29–32). Calcium and vitamin D supplementation has demonstrated modest reductions in stress fracture risk in certain controlled settings (22), but these findings have not been replicated (33). The most recent Health of the Force report, which includes incidence of injury data from 2018 to 2022, describes a steadily increasing incidence of overuse injuries (34). Bone stress injury incidence, specifically, remains orders of magnitude greater during basic military training, a period of 8 to 13 weeks of heightened physical activity at the start of military service, than during continued service (35–38).

Therefore, in this narrative review, we focus on the first several weeks of military service and training. We critically examine the historical evolution and current state of nutritional strategies for bone stress injury prevention in military populations, and we ask whether these strategies adequately address bone stress injury risk. We synthesize evidence from bone mechanobiology and nutritional science to examine whether past strategies have produced sustained reductions in bone stress injury incidence. Finally, we identify mechanistic and novel nutrition research opportunities that may support a more precise, robust, integrative nutritional framework capable of reducing bone stress injury prevalence in the military.

## Methods

The evidence in this narrative review was informed by primary military studies, systematic and narrative reviews, consensus and position statements, military surveillance and technical reports, Department of Defense and Service-level policies and guidance, National Academies reports, and selected historical sources. Primary military evidence included prospective and retrospective cohort studies, randomized and non-randomized interventions, and longitudinal studies conducted in U.S. and international military populations. We also used grey literature, including military reports, surveillance data, and policy documents to characterize the historical and contemporary burden of musculoskeletal injury (MSKI), military dietary requirements, nutrition policy, and implementation of injury- and nutrition-related programs. Searches combined terms related to military personnel and training (e.g., “military,” “recruit,” “basic training”), MSKI and bone health (e.g., “musculoskeletal injury,” “bone stress injury,” “stress fracture,” “bone mineral density,” “bone metabolism markers”), and factors relevant to injury prevention and military nutrition policy (e.g., “energy availability,” “calcium,” “vitamin D,” “protein,” “iron,” “nutrition,” “sleep,” “physical activity,” and “exercise”). We also examined reference lists of relevant articles and reviews to identify additional studies pertinent to the scope of the review. We considered only English-language studies.

When military-specific evidence was unavailable or insufficient to evaluate a potentially relevant intervention or biological mechanism, we considered evidence from non-military populations. We prioritized studies of athletes and other physically active populations when their exercise or mechanical-loading exposures were analogous to aspects of military training. We used evidence from populations less directly representative of service members, including older adults, primarily to establish biological mechanisms or to address questions of intervention efficacy that the available military literature could not resolve. We interpreted findings from these populations as indirect evidence and did not assume they generalized directly to military personnel. Accordingly, we placed greater interpretive weight on military-specific studies and studies with direct injury or fracture outcomes than on studies conducted in indirect populations or those reporting structural, biochemical, or mechanistic surrogate outcomes. Consistent with a narrative review approach, the literature search was not intended to be exhaustive or systematic, we selected and interpreted literature according to its relevance to the scope and objectives of the review and its ability to inform the relationships among nutrition, bone health, and MSKI in military populations.

### History of nutrition policy

To understand how nutrition policy has shaped the existing landscape with respect to musculoskeletal and bone stress injury, we must first consider its historical foundations as recognition that nutrition underpins military readiness dates back more than a century. During World War I, the Office of the Surgeon General established a Division of Food and Nutrition to inspect food preparation, improve sanitation in training camps, and evaluate ration adequacy for sustaining health and performance (39). During World War II, the U.S. Army expanded these activities substantially, working with civilian scientists through the Office of Scientific Research and Development to develop improved operational rations and to evaluate energy and nutrient requirements under combat conditions (39).

Following World War II, military nutrition policy became increasingly formalized. In 1947, the Department of the Army implemented Army Regulation 40–250, establishing the dietary standards for garrison and field rations and initiating the first use of “Minimum Nutrient Intake” for servicemembers (40). Later, the first tri-service regulation, now Army Regulation 40–25, defined nutrition and menu standards for garrison feeding and operational environments and has been periodically updated to reflect advances in nutritional science and changing operational demands (41). These efforts contributed to the development of the Military Recommended Dietary Allowances (MRDAs), revised in 1985 (41, 42), reflecting changes made to the civilian Recommended Dietary Allowances developed by the National Research Council, incorporating military-specific considerations such as increased physical activity, environmental stress, and operational demands (43, 44). However, limitations in applying civilian-derived standards to military populations became apparent. Reports conducted by the Committee on Military Nutrition Research in 1995 and again in 2006, concluded that military-specific dietary reference standards were needed because operational environments substantially alter energy expenditure and nutrient requirements (43, 44).

These recommendations led to the establishment of the Military Dietary Reference Intakes (MDRIs) in 2001 and revised in 2017, which replaced the MRDAs, and provided quantitative nutrient intake recommendations specifically tailored to military personnel. The MDRIs incorporated environmental stressors, physical activity levels, and mission demands, and formed the scientific basis for menu planning, nutrition education, and ration development across the services (45). Complementary guidance, such as the Nutritional Standards for Operational and Restricted Rations, was developed to ensure that rations met the elevated energy and nutrient demands associated with military operations (45).

In response to rising injury rates, a report published by the Armed Forces Epidemiological Board at the request of the Office of the Surgeon General of the Army, concluded that injuries impose a greater burden on the health and readiness of the U.S. Armed Forces than any other category of medical complaint during peacetime and combat (46). These findings coincided with growing recognition of nutrition's role in bone health. A 1997 workshop convened by the Subcommittee on Body Composition, Nutrition, and Health of Military Women, a subcommittee of the Committee on Military Nutrition Research, highlighted the high incidence of stress fractures in military populations, specifically in women, and identified inadequate calcium intake and energy restriction as modifiable risk factors (9). This workshop contributed to a broader shift toward integrating nutrition into injury prevention and performance optimization efforts, reflected in subsequent health promotion initiatives including the development of the dining facilities Go for Green program in 2008 (47).

More recently, military nutrition policy has been integrated into a comprehensive framework. The establishment of the Department of Defense Nutrition Committee in 2011 formalized coordination across the services to standardize nutrition policy and guidance (48). In 2020, Field Manual 7–22 formally integrated nutrition, physical training, and recovery as core components of the Army's Holistic Health and Fitness system, recognizing nutrition as essential to sustaining readiness, reducing injury risk, and optimizing performance (49). This marked the first time clinical nutrition expertise was systematically embedded within operational units as a core readiness capability rather than a purely medical or sustainment function.

Although these policy developments progressively strengthened military nutrition standards, evidence that their implementation translated into improved nutritional status or reduced bone stress injury remains considerably more limited. Establishment of military-specific nutrient standards has not consistently translated into achievement of those standards during training. In a recent multicenter study of 587 male and female Army trainees, vitamin D remained the most prevalent shortfall nutrient: 55% of men and 70% of women consumed less than one-third of the MDRI, and fewer than half of women met the MDRIs for calcium, iron, magnesium, potassium, fiber, vitamin E, and α-linolenic acid (50). Recent Defense Medical Surveillance System data show that clinically diagnosed vitamin D deficiency remained relatively stable among active-component personnel from 2018 through 2022, with an average annual prevalence of 2.2% (51). This prevalence is substantially lower than that of the U.S. general population, but the low rate may reflect limited screening for vitamin D deficiency among service members rather than lower true prevalence, since the U.S. Preventive Services Task Force does not recommend universal screening for vitamin D, and TRICARE does not cover screening for asymptomatic individuals.

Go for Green was introduced in 2008. In a 2014 evaluation, U.S. Army soldiers who reported using the program's nutrition labeling system consumed a lower proportion of total energy from fat than non-users (32% vs. 36%), though fewer than half of the nearly 300 soldiers surveyed reported using the nutritional labels regularly (52). A subsequent evaluation reported that approximately two-thirds (66%) of soldiers used Go for Green labels at least once per day (53). Implementation, however, has been inconsistent, and an Army assessment cited in that report found that 41% of installations visited did not meet nutrition standards (54).

Implementation of the Army's Holistic Health and Fitness (H2F) system has improved several measures of soldier readiness. In a matched analysis of 56 active duty brigades, brigades resourced with embedded H2F Performance Teams showed a 61% reduction in the odds of MSKI referral, alongside improvements in physical fitness, body composition compliance, behavioral health outcomes, and duty availability between fiscal years 2019 and 2023 (55). However, H2F fielding was progressive, and most teams reached only approximately 30% staffing by fiscal year 2021 and >75% staffing by fiscal year 2022. Subsequent operational reports have identified substantial variability in staffing, integration, soldier engagement, and utilization across units (54). Although nutritional readiness is a core H2F domain, available evaluations have not directly assessed dietary intake, nutrient adequacy, energy availability, or bone stress injury outcomes, which precludes attributing observed improvements in MSKI or readiness to H2F's nutritional component.

Together, these developments reflect more than a century of evolving military nutrition policy, progressing from basic ration adequacy during wartime to modern, evidence-based performance nutrition systems designed to support operational effectiveness, injury prevention, and long-term force readiness. Military surveillance incidence estimates vary across studies and surveillance periods; differences in populations, diagnostic definitions, coding practices, and surveillance methods preclude interpreting these data as evidence of a consistent temporal change over time (56–58). However, with respect specifically to MSKI prevention, and bone stress injury in particular, available evidence does not demonstrate that these efforts have produced sustained reductions in incidence as available evidence indicates that bone stress injuries continue to impose a substantial and disproportionately high burden during the start of military service, particularly among female trainees, despite longstanding efforts to mitigate injury risk. Importantly, prevention of bone stress injury is only one component of the broader objectives of military nutrition policy, and the absence of demonstrated reductions should not be interpreted as evidence that these policies have failed to achieve their other intended outcomes.

### Policy in practice: existing nutritional interventions

While many studies have identified risk factors for MSKI (16, 59–61), comparatively few have evaluated targeted nutritional interventions designed to modify those risks. A recent scoping review by Bullock et al. (5) of injury mitigation programs in military populations identified 90 controlled studies evaluating injury prevention strategies; only 3 examined nutritional interventions, compared with approximately 38 exercise-based and 33 equipment-based interventions. This imbalance highlights a critical gap in the evidence base underpinning military injury prevention policy. The following section synthesizes nutrition-based strategies implemented or tested within military populations and critically examines their evidentiary basis and limitations (Tables 1, 2).

**Table 1:** Summary table including chronologically ordered studies examining bone stress injury incidence or general musculoskeletal injury.

References	Population (*N*)	Study design & duration	Intervention & comparator (*if applicable*)	Principle bone/fracture outcomes	Primary Findings	Limitations
BONE STRESS INJURY OR STRESS FRACTURE INCIDENCE						
Milgrom et al. (14)	Male Israeli military recruits (295)	14-wk prospective observational cohort		Stress fracture	High stress-fracture incidence during recruit training.	No intervention/comparator; observational; exposure/risk factors not experimentally controlled.
Schwellnus and Jordaan (78)	Male military recruits (1,398)	9-wk randomized clinical trial	Ca 500 mg/d vs. control	Stress fracture; overuse injuries	No significant reduction in stress injuries.	Few fracture events.
Lappe et al. (22)	Female U.S. Navy recruits (5,201)	8-wk randomized, double-blind, placebo-controlled trial	Ca 2,000 mg/d + vitamin D 800 IU/d vs. placebo	Stress fractures	20% lower stress-fracture incidence with supplementation.	High attrition; fracture ascertainment depended on symptomatic presentation;
Merkel et al. (99)	Female Israeli combat recruits (438)	4-month prospective cohort		Stress fracture	Female recruits who sustained a fracture had significantly lower levels of serum iron and iron saturation.	Low incidence of fracture; none observed in male or non-combat comparator.
Yanovich et al. (98)	Male and female Israeli recruits (100)	16-month prospective observational cohort		Stress fracture; iron status	Female combatant fracture group had more anemia/iron-deficiency anemia.	Large attrition; few fracture including none in men, limiting sex comparisons.
Barnes et al. (178)	Female U.S. Air Force trainees (supplement program *n* = 4,303; historical control *n* = 4,051)	8.5-wk cohort-based pilot study with season-matched historical control	Prenatal vitamin (27 mg iron, 200 mg Ca, 400 IU vitamin D/d) offered during BMT vs. historical control	Stress fracture	Prenatal vitamins did not significantly reduce stress fractures or medical attrition.	Historical control; supplement compliance unknown.
Davey et al. (23)	Male Royal Marine recruits (1,082)	32-wk prospective observational cohort		Stress fracture	Lower baseline 25(OH)D (50 nmol/L) was associated with greater fracture risk.	Blood not drawn at time of injury.
Moran et al. (72)	Male Israeli Defense Forces combat recruits (73)	6-month prospective cohort study		Bone stress injury	CHO, Ca, and vitamin D intakes for the BSI group at induction were 55.1 – 67.9% less than Nutritional Standards requirements; No differences in hemoglobin, folic acid, calcium, iron, transferrin, ferritin, 25(OH)D and PTH between groups.	Dietary intake based on recall; low prevalence of vitamin D deficiency among recruits; zinc not measured.
Fredericson et al. (158)	Female collegiate distance runners (historical n=56; intervention n=78)	7-y multisite retrospective, prospective pilot, and interventional study	Nutrition education vs. historical phase	Bone stress injuries	No reduction in BSI rates between phases; *post hoc* analyses determined decline in trabecular-rich BSI but not cortical.	Changes in dietary intake were not directly measured; potential recall bias; early stop due to COVID-19.
Carswell et al. (64)	Male and female British Army recruits (2,166)	12-wk prospective cohort		Lower body overuse musculoskeletal injury and bone stress injury	Lower body BSI incidence higher for recruits within the lowest quartile of 24,25(OH)_2_D; Greater conversion of 25(OH)D to 24,25(OH)_2_D relative to 1,25(OH)_2_D was associated with lower BSI incidence; Neither serum 25(OH)D nor 1,25(OH)_2_D related to injury risk when examined in isolation.	Concentration of metabolites measured during first week of training, not at time of injury.
15.6-7.4,-74.8690ptO'Leary et al. (97)	Male and Female British Army trainees (2277)	13-wk prospective cohort		Lower limb overuse injury; lower limb stress fracture (including hip/pelvis)	Lower ferritin associated with slower run time, sustaining a lower limb overuse injury, lower aBMD, and higher βCTX and PINP controlling for sex; No marker of iron status was associated with lower limb stress fracture;	Did not measure hepcidin or transferrin; male and female trainees completed different training courses; did not control for plasma volume nor physical activity before blood samples; dietary iron intake not assessed; menstrual status in female recruits not assessed.
GENERAL MUSCULOSKELETAL INJURY						
McGinnis et al. (100)	Male U.S. Army recruits (2,174)	9-wk (BCT) or 14-week (OSUT) randomized controlled trial	Protein/carbohydrate supplementation vs. non-supplemented soldiers	Musculoskeletal injuries/missed training	Supplementation was associated with fewer MSIs and missed training days.	Comparator data from non-supplemented soldiers was accessed from historical data from whom dietary recalls were not collected; MSKIs not radiographically confirmed; unknown compliance.
Grier et al. (163)	Male and female U.S. Army Special Forces soldiers (7,576)	Retrospective analysis of military records		Musculoskeletal injuries	Soldiers who slept ≤ 4 hrs. were 2.35 times more likely to suffer a MSKI compared with those who slept eight ≥ 8 hrs.; older men had higher risk of a MSKI than their younger men across all sleep duration groups.	Under representation of women; population 95% male.
Dijksma et al. (126)	Male Royal Netherlands Army Air recruits (48)	7–12-wk pre-training conditioning program in low-fit recruits vs. controls (entered BMT without delay).		Clinical MSKI incidence; training-related MSKI dropout; total attrition; lost training days.	Intervention improved run performance; No significant difference in injury incidence or dropout rate.	Small sample, extremely high attrition, substantial missing data, limited statistical power and precision.
Sekel et al. (71)	Male and female Marine Officer Candidates School trainees (227)	10-wk prospective cohort		Lower extremity overuse musculoskeletal injury	Increased baseline hepcidin was associated with an increased likelihood of suffering a MSKI in male trainees but not female; No other micronutrient parameters were predictive of injury.	Trainees were not exclusively fasted nor was physical activity controlled for prior to blood sampling; PTH, hemoglobin, and soluble transferrin receptor not measured.
Foulis et al. (19)	Male and female U.S. Army recruits (2,988)	10-wk BCT and 6-week post-BCT prospective cohort		Factors in MSKI risk during military training	Low vitamin D levels were risk factors for MSKI in the total cohort and male-specific models but not in the female-specific model; Higher levels of serum vitamin D (OR,1.00; 95%CI, 0.99–1.01), iron (OR, 1.00; 95% CI, 1.00–1.00), or ferritin (OR, 1.00; 95% CI, 1.00–1.00) were associated with lower MSKI risk.	Did not evaluate change in variables and their relationship with MSKI.

BCT, basic combat training; Ca, calcium; CHO, carbohydrate; CI, confidence interval; d, day; g, gram; h, hour; IU, International Units; kg, kilogram; mg, milligrams; MSKI, musculoskeletal injury; OSUT, One Station Unit Training; PTH, parathyroid hormone; P1NP, Procollagen 1 N-terminal propeptide; U.S., United States; wk, week; y, year; 25(OH)D, 25-hydroxy-vitamin D; 24,25(OH)_2_D, 24,25-dihydroxyvitamin D; 1,25(OH)_2_D, calcitriol; β-CTX, Beta C-terminal telopeptide cross-links of type 1 collagen.

**Table 2:** Summary table including chronologically ordered studies examining surrogate outcomes related to bone stress injury.

References	Population (*N*)	Study design & duration	Intervention & comparator (*if applicable*)	Principle bone/fracture outcomes	Primary Findings	Limitations
BONE STRUCTURAL, BIOCHEMICAL, OR MECHANISTIC SURROGATE OUTCOMES						
Ihle and Loucks (111)	Sedentary young women (29)	9-day prospective cohort	Restricted energy availability ranging between 10–30 kcal/kg LBM/d	Bone and calcium metabolism markers	PICP declined linearly with energy availability; urinary NTX only raised at lowest energy availability.	Specific macronutrient restriction not assessed.
Gaffney-Stomberg et al. (133)	Male and female U.S. Army basic training recruits (243)	9-wk randomized, double-blind, placebo-controlled trial	Ca 2,000 mg/d + vitamin D 1,000 IU/d vs. placebo	PTH, 25(OH)D, pQCT BMD/geometry	Supplementation attenuated PTH elevation and improved select tibial bone measures; greater decline in area and circumference observed in supplemented group.	pQCT resolution unable to discern microarchitectural parameters; analyses not stratified by sex.
Hughes et al. (179)	Male U.S. Army Ranger trainees (*n* = 22; follow-up subset *n* = 8)	8-wk prospective pre-post study; subset reassessed 2–6 wk after training	U.S. Army Ranger Training with high energy expenditure, energy restriction, and sleep deprivation; no non-training comparator	BAP, osteocalcin, CTX, TRAP5b, PTH, calcium, 25(OH)D	Bone formation was suppressed and resorption increased: BAP decreased 22.8%, osteocalcin decreased 21.0%, and TRAP5b increased 57.5% from pre- to post-training. Osteocalcin and TRAP5b returned to baseline during recovery, whereas BAP remained suppressed.	Small male-only cohort; multiple simultaneous stressors preclude isolation of energy deficit effects; no control group or fracture endpoint; recovery data available in only 8 participants.
Sale et al. (103)	Physically active, healthy Male adults (10)	7-d randomized crossover exercise study	CHO feeding during running vs. placebo	CTX/P1NP; bone metabolic response	CHO attenuated the exercise-related increase in bone resorption.	Very small sample; small magnitude of effect.
Townsend et al. (104)	Male adult endurance runners (10)	Acute randomized/crossover exercise study	Postexercise carbohydrate + protein vs. control	Bone turnover markers	Nutrition modified the acute bone metabolic response.	Uncertain implications in the attenuation of resorption marker concentrations on injury risk.
Papageorgiou et al. (114)	Physically active young men and women (22)	10-day crossover design vs. control	low energy availability 15 kcal·kgLBM^−1^·d^−1^	Bone and calcium metabolism markers	In women: βCTX AUC significantly higher and P1NP AUC significantly lower in restricted group; In men: No change by group; No differences by sex observed or in calcium or regulatory hormones.	Specific macronutrient restriction not assessed.
Sherk et al. (83)	Competitive male cyclists (50)	Acute randomized controlled trial	Ca 1,000 mg 30-mins pre-exercise vs. placebo	iCa, PTH, CTX	Ca attenuated the decline in iCa; no significant differences observed in CTX or PTH.	Some men met criteria for lone bone mass.
Rahme (180)	Male and female overweight elderly individuals (257)	12-month randomized controlled trial	1,000 mg of elemental Ca citrate/day, and the daily equivalent of 3,750 IU/day or 600 IU/day of vitamin D3	Bone metabolism markers; aBMD	No significant differences in percent change aBMD between groups at any skeletal site.	No placebo arm.
Kohrt et al. (84)	Young cycling trained men (11)	Acute randomized crossover	IV Ca clamp vs. saline during 60-mins cycling bouts	PTH, CTX	Maintaining iCa attenuated exercise-induced PTH and CTX responses; No difference in P1NP response.	Very small male sample; acute mechanistic design.
Hughes et al. (37)	Female U.S. Army recruits (90)	8-wk prospective cohort		Tibial microarchitecture by HR-pQCT	Training produced changes in distal tibial density/microarchitecture consistent with new bone formation.	No non-training control.
O'Leary et al. (74)	Male British Army military infantry recruits (42)	13-wk prospective cohort		Tibial macro/microstructure by HR-pQCT; bone metabolism markers	Training increased trabecular/cortical density and size; resorption decreased; no changes in P1NP, PTH, Ca.	Scanning limited to distal tibia; pre/post design precludes interpretation of temporal changes in bone.
Gaffney-Stomberg et al. (25)	Male and female U.S. Marine initial training recruits (197)	12-wk randomized, placebo-controlled trial	Ca 2,000 mg/d + vitamin D 1,000 IU/d vs. placebo	Tibial pQCT density/strength, 25(OH)D	BAP and TRAP5b decreased over training in Ca + D group but not in placebo; Largely null effects of supplementation on bone tissue; 25(OH)D was positively associated with change in tibial metaphyseal strength.	Pre/post study design may have precluded full resolution of biomarker response.
Sefton et al. (101)	Male U.S. Army initial entry training soldiers (89)	9-week placebo-controlled, double-blind study	CHO 63.4 g or 80% whey protein concentrate once/day	Bone metabolism markers; vitamin D; Ca	No influence of supplementation on bone or calciotropic biomarkers across training.	No randomization; compliance unknown.
O'Leary et al. (112)	Male British Army infantry soldiers (30)	8-wk non-randomized controlled trial	Supplementary energy (5.1 MJ·d^−1^) vs. control group (habitual diet)	Bone metabolism markers; reproductive, thyroid, and metabolic status markers	BAP decreased in controls and increased in the supplemented group; no effect of group on PINP or βCTX; Estradiol, total and free testosterone, and IGF-1 decreased over time.	Intervention and control groups completed courses at different times of the year; no direct measurement of energy intake or expenditure.
Wherry et al. (85)	Male and female older adults (12)	Acute randomized crossover study	Ca infusion vs. saline during 60-min walking bouts	PTH, CTX	Ca attenuated exercise-induced iCa decline and subsequent PTH and CTX increases.	Very small sample; bone formation not assessed.
O'Leary et al. (76)	Male and female British Army soldiers (177)	14-wk prospective observational cohort		Tibial density, geometry, strength by pQCT	Training increased tibial size, density, and strength; P1NP increased; βCTX and sclerostin decreased; largely similar adaptations by sex.	No non-training control.
O'Leary et al. (102)	Male British Army Officer cadets (44; 14 women included as control)	36-h military field exercise	Habitual diet in women and male controls; additional 46.6 g protein/d in supplemented men during an energy-deficit field exercise	β-CTX, P1NP, PTH, 25(OH)D and endocrine markers	P1NP decreased after the field exercise and remained suppressed at 96-h recovery, while PTH increased postexercise; responses were similar in women and male controls. β-CTX did not significantly change. Additional protein in men did not alter any bone-metabolism marker.	Protein supplementation evaluated only in men; high volume of protein consumed in control group.
Coombs et al. (136)	Civilian and servicewomen (47)	Acute randomized crossover study	Ca 1,000 mg 60 min before load carriage (20 kg) vs. control	Bone Ca balance, PTH, bone metabolism markers	Ca prevented the decrease in iCa, suppressed the increase in PTH and decreased bone resorption; sclerostin increased post exercise independent of calcium status.	Ca isotope method precluded ability to determine amount of ca absorbed during exercise; unblinded; habitual ca intake not assessed.

AUC, area under the curve; Ca, calcium; CHO, carbohydrate; CTX, C-terminal telopeptide cross-links of type 1 collagen; d, day; g, gram; h, hour; H2F, Holistic Health and Fitness; iCa, ionized calcium; IU, International Units; kg, kilogram; mg, milligrams; LBM, lean body mass; MJ, megajoules; pQCT, peripheral quantitative computed tomography PTH, parathyroid hormone; P1NP, Procollagen 1 N-terminal propeptide; UL, upper intake level; U.S., United States; wk, week; 25(OH)D, 25-hydroxy-vitamin D; β-CTX, Beta C-terminal telopeptide cross-links of type 1 collagen BAP, Bone alkaline phosphatase; TRAP5b, Tartrate-resistant acid phosphatase 5b.

### Calcium and vitamin D

Vitamin D status has been linked to stress fracture risk among new male (23, 62–64) and female (22, 64–66) military trainees. Moreover, low serum 25-hydroxy-vitamin D (25[OH]D) has been associated with longer recovery time post-fracture; those who are vitamin D deficient (< 25 nmol/L) require 22.3% more recovery time compared with those who are vitamin D sufficient (67). Despite the established association between vitamin D status and injury risk, longitudinal observational studies examining changes in 25(OH)D during basic military training have produced inconsistent findings, with prior work demonstrating diminished (23, 68–71), stagnant (72, 73), or improved (74, 75) 25[OH]D concentrations resulting from military training, with additional variability observed by sex (73, 76), season (70), and race (68, 77). For example, male Estonian conscripts (69) and Royal Marines (23) exhibited substantial (30.7 to 48.2%) declines in 25(OH)D concentrations over the course of 10 to 32 weeks of training despite low baseline concentrations. Male British Army trainees, however, increased in 25(OH)D concentrations by 18.7% over a similar time course (74, 76), while women undergoing the same training decreased (76). Male and female Israel Defense Forces combat trainees exhibited no change (72) or a decline in men only (73) despite significant declines in serum calcium and parathyroid hormone concentrations during the first 2 months of training.

The most frequently cited successful trial, a randomized, placebo-controlled trial in female U.S. Navy trainees, reported a 20% reduction in stress fracture incidence with calcium (2,000 mg/d) and vitamin D (800 IU/d) supplementation during basic military training (22). This study was not without limitations: it was conducted exclusively in female trainees with exceptionally low habitual calcium intake (300 mg/day, equivalent to one 8-ounce serving of dairy milk), which may have amplified the observed benefit. Furthermore, the absolute risk reduction was modest, and similar reductions have not been consistently replicated in other military populations (33). For instance, male trainees undergoing a 9-week physical training program with high dietary calcium intake (>800 mg/day) who received 500 mg of supplemental calcium per day showed no detectable difference in bone stress injury incidence when compared to non-supplemented controls. Importantly, very few stress fractures were recorded over the course of the study, potentially precluding the analytical ability to ascertain significant differences between groups (78).

In 2018, the Combat Feeding Directorate (Natick Soldier Research, Development and Engineering Center) in partnership with the U.S. Army Research Institute of Environmental Medicine's Military Nutrition Division developed the Performance Readiness Bar, a daily snack product fortified with 850–1,100 mg of calcium and 1,400–2,000 IU of vitamin D (79). When a snack bar similar to this operational ration containing 1,032 mg calcium carbonate and 546 IU vitamin D_3_ was delivered twice per day throughout 9-weeks of basic military training to a mixed-sex cohort, supplementation both increased circulating concentrations of calcium and maintained parathyroid hormone during training (24). Growing experimental evidence suggests that the disturbance to calcium homeostasis during exercise results in clinically-meaningful axial bone loss (80, 81) and therefore attenuating this disturbance may mitigate the catabolic effect of exercise on bone (82–85). Concurrent imaging data by peripheral quantitative computed tomography (pQCT) showed that the fortified bar also improved total volumetric bone mineral density and cortical thickness at the ultradistal and distal tibia, respectively (24). The authors postulate these adaptations resulted from supplementation suppressing bone remodeling, reducing demineralization (24). Though these findings are potentially consequential, as cortex thickness dramatically improves the mechanical competence of loaded bone (86), the resolution of the pQCT precludes quantification of microarchitectural parameters, which independently contribute to the increases in tibial strength following military training (37). Furthermore, imaging was limited to the distal tibia, a predominantly trabecular region subjected primarily to compressive loading. This may be an important limitation in military populations, in whom the tibial diaphysis is exposed to predominantly bending stresses and may be more clinically relevant to injury (87). In a subsequent study, a snack bar containing 945–1,123 mg calcium and 429 to 518 IU of vitamin D_3_ was issued to male and female trainees during U.S. Marine Corps basic military training, but no effect was observed on tibial adaptations as measured by pQCT (25). While these results may suggest that greater doses are necessary for skeletal benefit, evidence from longer-duration supplementation trials are inconsistent (180). For example, in a 3-year randomized clinical trial of healthy adults, supplementation with 4,000 or 10,000 IU/day of vitamin D3 resulted in greater reductions in high-resolution peripheral quantitative computed tomography radial volumetric BMD compared with 400 IU/day, while 10,000 IU/day also resulted in greater reductions in tibial volumetric BMD (88). While mechanistically plausible, the efficacy of the Performance Readiness Bar, and similar fortified snack bars, for preventing bone stress injuries has not been determined.

### Iron

Both elevated (i.e., iron overload conditions such as hemochromatosis) and low (i.e., iron deficiency with or without anemia) iron status are associated with weakened bones, suggesting that bone homeostasis requires iron status maintained within a narrow physiological range (89). Current evidence suggests that both high and low iron status promotes bone loss by modulating the differentiation and activity of osteoclast and osteoblasts (90). Elevated iron also exerts deleterious effects on bone. Guggenbuhl et al. (91) investigated the role of modestly elevated iron concentrations on the in vitro growth of hydroxyapatite crystals, the most abundant (60%) inorganic component of bone. They found that even modestly elevated iron inhibits crystal growth, resulting in decreased crystal size and decreased crystal quality. This is consequential, as decreased crystal quality may reduce bone strength (92). Iron deficiency is highly prevalent among military trainees, particularly women, with studies reporting that 20–50% of female trainees exhibit depleted iron stores or iron deficiency, with or without anemia, when starting basic military training, and that lower iron status is associated with reduced aerobic performance, increased fatigue, and higher attrition risk (93–95). As a result, the U.S. Army implemented a multivitamin with iron program beginning in September 2015 for female trainees. An injury surveillance evaluation compared injury rates before and after program implementation and reported a significant reduction in overall injury incidence among female trainees (−3%) following the program's introduction; male trainees, who did not receive the supplement, showed no comparable reduction (6). However, supplementation was voluntary, and supplement intake, age, fitness level, and body composition were not controlled across the year-long observation period. Therefore, while supportive, these findings do not clarify whether iron supplementation reduced BSI incidence in female trainees (96).

Observational studies examining iron status and bone stress injury risk have produced inconsistent findings. Several investigations in military populations found no association between hemoglobin, soluble transferrin receptor, or red cell distribution width and stress fracture incidence (72, 97). In contrast, studies focused on female trainees have reported that those who developed stress fractures had lower serum ferritin, lower transferrin saturation, and higher prevalence of iron deficiency or anemia compared with uninjured controls (97–99). In a large sample of male and female British Army trainees, lower baseline ferritin was modestly associated with lower limb overuse injury but not stress fracture during training (97). Finally, in male but not female Marine trainees undergoing 10-weeks of Officer Candidates School, elevated hepcidin concentrations, indicative of increased iron sequestering, predicted lower extremity overuse injury during training (71).

### Macronutrient intake: protein and carbohydrate

Few interventions targeting macronutrient intake during basic military training have been evaluated as potential countermeasures to injury. In a controlled trial of male U.S. Army trainees, McGinnis et al. (100) compared daily supplementation with whey protein or carbohydrate, consumed once or twice per day. Trainees consuming two daily servings of either supplement had lower MSKI incidence, fewer limited-duty profiles, and fewer missed training days compared with historical non-supplemented controls; However, one daily serving did not reduce injury risk. In a subsequent study, Sefton et al. (101) examined bone metabolic responses to whey protein or nearly calorie-matched carbohydrate supplementation during 9-weeks of U.S. Army training. Although vitamin D increased across training, no responses to supplementation, including in procollagen 1 N-terminal propeptide (P1NP), C-terminal telopeptide cross-links of type 1 collagen (CTX), vitamin D, nor calcium differed between groups. Similarly, in a 36-h field exercise performed by British Army officer cadets in energy deficit (~2,000 to 3,000 kcal·d-1), protein supplementation did not attenuate the reduction in P1NP nor the transient rise in PTH, suggesting that targeted macronutrient supplementation was not protective during arduous training (102).

These findings in military personnel differ from randomized crossover studies in non-military populations that collectively suggest carbohydrate availability may influence acute bone turnover responses to exercise. For example, Sale et al. (103) reported that carbohydrate ingestion before, during, and after prolonged running attenuated acute increases in CTX and P1NP, compared with placebo. However, these effects were transient and did not persist beyond the immediate recovery period. Similarly, Townsend et al. (104) found that immediate post-exercise ingestion of carbohydrate combined with protein improved acute bone turnover balance following exhaustive running. However, across these studies, the observed biochemical changes occurred without evidence of corresponding improvements in bone structural adaptation or injury risk, which were not assessed (105).

### Energy availability

Low energy availability suppresses bone formation, alters hormonal signaling, and may impair skeletal adaptation to training (15, 106). Weight-bearing activity alone does not appear sufficient to overcome the detrimental effects of low energy availability on bone, as demonstrated by impaired bone density, microarchitecture, and strength in athletes, including both men (107) and women (108–110). In a seminal controlled feeding and exercise study, Ihle and Loucks (111) experimentally reduced energy availability in healthy, habitually sedentary young women of normal body composition and observed dose-dependent suppression of osteocalcin and procollagen type I carboxy-terminal propeptide, along with increased bone resorption as indicated by urinary N-telopeptide. These changes occurred even at moderate levels of low energy availability (below ~30 kcal/kg of lean body mass), demonstrating that inadequate energy availability rapidly disrupts bone turnover balance principally by suppressing bone formation (111). In a recent non-randomized controlled trial, O'Leary et al. (112) determined that providing supplementary energy (5.1 MJ·d-1) to attenuate the energy deficit observed during an 8-week military combat course increased bone formation marker bone alkaline phosphatase in male soldiers but did not prevent declines in circulating insulin-like growth factor 1 or testosterone (113), suggesting that although additional energy may partially support bone formation, it may be insufficient to preserve the anabolic hormonal milieu required for optimal skeletal adaptation during strenuous military training (179).

Short-term energy deficits also produce measurable effects. Experimental studies demonstrate that as few as 5-days of low energy availability (defined as dietary energy intake minus exercise energy expenditure, adjusted for muscle mass) can uncouple bone turnover, increasing bone resorption markers by approximately 30–35% while suppressing bone formation markers, which may indicate an acute shift toward net bone loss (114). However, evidence that correcting low energy availability reduces bone stress injury risk in military populations remains extremely limited (115) and to our knowledge, no study has directly examined the effect of energy availability on bone health during basic military training. Additionally, few studies have compared the bone metabolic response to energy deficits between males and females, despite athlete literature suggesting sex-specific endocrine and metabolic responses to energy deficit (102, 116).

## Discussion

The “Funnel Effect,” as conceptualized by Jepsen and colleagues, describes how numerous biological and genetic pathways converge over time to shape bone structure and, ultimately, fracture susceptibility. Rather than arising from a single deficit, bone fragility reflects the cumulative narrowing of multiple upstream influences, including bone geometry, material properties, and tissue-level organization, that collectively determine whole-bone strength (117). Fracture risk is therefore best understood as the product of interacting determinants rather than any single exposure. Importantly, these factors rarely operate in isolation; their combined and potentially synergistic effects complicate both interpretation and intervention. A forward-leaning strategy must therefore extend beyond solely dietary supplementation with micronutrients or altering macronutrient intake to address timing, implementation, and the broader physiological context in which skeletal adaptation to training occurs.

### Injury timing

Basic training lasts weeks, but building strong troops takes years.

General (Ret.) Richard B. Myers US Air Force, 15^th^ Chairman of the Joint Chiefs of Staff (54).

Trainees experience bone stress injuries within just weeks of beginning basic military training, coinciding temporally with an increase in osteoclastic bone resorption, the first stage of a coupled remodeling response that is estimated to last for approximately 6 to 7 weeks (1). Targeted remodeling repairs accumulated microdamage while the residual excavated resorption cavity transiently reduces bone volume, increases porosity, and consequently reduces bone stiffness (118), weakening bone (11, 12). As hypothesized by Hughes et al. (12) and others (119–121), newly activated remodeling cycles remain in early stages when negative bone balance can contribute to a cycle of increased strain (1) and accumulation of microdamage upon continued loading until a bone stress injury occurs.

Prospective studies demonstrate that stress fractures frequently occur early in training. In female U.S. Navy basic military trainees, median time to stress fracture diagnosis was just 36 days (22). Epidemiologic data consistently shows that the majority of stress fractures occur during the first several weeks of military service (122). In Israeli Defense Force trainees, 10% of symptomatic stress fractures had pain onset before basic military training, while 53% began prior to week 4 and 22% during weeks 5 through 8 (14). More recent U.S. Army data similarly show sharp increases in diagnoses during weeks 3–4 of basic military training, peaking between weeks 5 and 8, with new cases continuing to emerge beyond the 20th week of military service (36). The occurrence of stress fractures within the first few weeks of training is difficult to reconcile with the proposed pathophysiological sequence of repetitive loading-induced fatigue damage, targeted remodeling, transient increases in porosity, and eventual structural failure, as these processes likely require longer than 1 to 2 weeks to develop. Plausible explanations include that recruits who abruptly changed the nature or intensity of exercise before starting basic military training began training in a state of negative bone balance or with pre-existing bone damage, delayed symptom reporting or diagnostic delay, anatomical differences, or variation in training exposure. External validation of these mechanistically plausible explanations is required.

Nearly 30 years ago, the Subcommittee on Body Composition, Nutrition, and Health of Military Women concluded that the duration of basic military training, which differs by branch of service, may be insufficient to fully develop physiological adaptations capable of reducing injury risk (9). Moran et al. (72) showed that dietary deficiencies present before basic military training began were associated with stress fracture development in male Israeli Defense Forces trainees. Strengthening bone by modeling and remodeling requires time. Given that meaningful changes in bone mass, structure, and mechanical competence generally require at least one year to develop (115, 123), nutritional strategies intended to reduce bone stress injuries during basic military training would be most effective when implemented well before training begins and sustained throughout military service.

Although the interval between medical qualification at the Military Entrance Processing Station (MEPS) and the start of basic military training currently varies widely, with some recruits shipping within days to a basic training site and others remaining in the Delayed Entry Program for months, a standardized pre-training period after MEPS would give all trainees sufficient time to progressively increase physical activity and, where indicated, receive targeted nutritional supplementation to better withstand the demands of military training. U.S. Navy Future Sailors (86% male) participating in an approximately 12-week Delayed Entry Program intervention that combined nutrition education and physical training supports the feasibility of this approach. Nutrition knowledge scores increased from 58.1% to 68.8%, while the intervention also identifed substantial inadequacies in energy, calcium, and vitamin D intake before training (124). Participants subsequently achieved a 92.4% basic training graduation rate, compared with approximately 87% from Recruit Training Command during the same period, and showed higher physical readiness scores than the 2021 historical average (78% vs. 58%). The study design limits attribution of these outcomes to the intervention, but the findings demonstrate the feasibility of using the Delayed Entry Program to address nutritional status and physical readiness simultaneously before basic training.

Lower fitness before starting basic military training is associated with greater subsequent injury risk, and pre-basic conditioning programs in U.S. Army recruits have improved physical fitness and shown some reduction in injury risk (125). However, a randomized controlled trial in the Netherlands Armed Forces found improved aerobic fitness but no reduction in MSKI or injury-related attrition following 7- to 12-weeks of pre-training conditioning (126). Additionally, in Royal Marine trainees, increased weight-bearing exercise before training was a significant risk factor for stress fracture occurrence (21). These studies indicate that the effectiveness, volume, and content of a standardized MEPS-to-training conditioning interval requires prospective evaluation before implementation.

### Nutrient timing and co-nutrient interactions

The osteogenic response to exercise is strongly influenced by the characteristics of mechanical loading, including magnitude, frequency, duration, and direction (127–129). Bone preferentially responds to non-uniform, complex, multi-directional strain patterns, rather than repetitive uniaxial loading, like those observed with running. Consistent with this principle, prospective epidemiologic studies in new military trainees with history of playing multi-directional sports exhibited 4 to 17% lower incidence of bone stress injuries during basic military training than trainees without (130). Even when exposed to regular weight-bearing exercise, the osteogenic response may be attenuated in the presence of suboptimal nutrition. Emerging evidence suggests that the timing of calcium supplementation, relative to exercise, has the potential to enhance bone adaptations to training. Exercise transiently perturbs systemic calcium homeostasis, reducing circulating ionized calcium and stimulating parathyroid hormone release, which in turn increases bone resorption to maintain calcium concentrations. It has been hypothesized that low habitual calcium intake (< 400 mg/day) is a contributing factor to the documented reductions in bone density at axial sites during basic military training (131) and less than half of female trainees meet the calcium requirements established in Army Regulation 40–25 (50). In the context of low dietary calcium intake, this compensatory response may increase skeletal calcium mobilization and contribute to cumulative bone stress. In competitive male cyclists, ingesting 1,000 mg of calcium either 20- or 30-min before exercise attenuated the post-exercise decrease in ionized calcium concentrations and blunted the increase in parathyroid hormone compared with placebo, but did not significantly reduce the increase in bone resorption (83, 132). The availability of calcium in the small intestine for absorption may reduce the extent to which bone calcium is mobilized during exercise (133). In contrast, taking untimed supplemental calcium with meals did not influence the trajectory of bone mineral density change over one year in competitive cyclists (134). Conversely, providing collegiate male athletes with supplemental calcium during practice for 1 year maintained dual-energy X-ray absorptiometry-derived bone mineral content (135). In a recent study in women, consuming a 1,000 mg calcium supplement 60-min before a 2 h bout of load carriage exercise attenuated the decline in ionized calcium concentrations, decreased parathyroid hormone release, and suppressed bone resorption compared to controls (136).

Existing micronutrient supplementation trials frequently fail to account for co-nutrient interactions. In order for calcium to exert a positive effect on bone, protein intake must be adequate (137, 138). Low protein diets (~0.7 g/kg/day) have been associated with elevated levels of parathyroid hormone and reduced intestinal calcium absorption, even with adequate calcium supplementation (139, 140). To date, few published calcium trials assessed or attempted to optimize protein intake (84, 133). Despite the importance of protein to the structural matrix of bone, in that protein comprises approximately half of bone volume and one-third of its mass, dietary protein intake has received much less focus than calcium and vitamin D for bone health (137, 141). Although dietary protein has historically been hypothesized to increase urinary calcium excretion and adversely affect bone through the acid–base balance theory, this concept has been largely disproven (142). Rather, calcium retention and bone mineralization are not negatively affected by increasing dietary protein intake (143, 144). Individual amino acids may also enhance skeletal calcium economy through distinct mechanisms. Lysine and arginine improve intestinal calcium absorption and increase skeletal calcium retention (145), while aromatic amino acids activate the calcium-sensing receptor (146), increasing fractional calcium absorption under conditions of low calcium intake, and suppress osteoclast differentiation (147). Aromatic amino acids have also been shown to preferentially stimulate insulin-like growth factor-1 secretion compared with branched-chain amino acids, providing an additional anabolic mechanism linking dietary protein quality to skeletal health (148, 149).

### Geographic injury clustering: targeted nutritional interventions to high-risk regions

A disproportionate injury burden originates from new military trainees from states in the southern region of the United States, highlighting an opportunity for targeted nutritional intervention. Large-scale U.S. Army training surveillance found that 34% of trainees from the South experienced at least one injury, significantly higher than all other regions, and eight of the ten states with the highest injury rates were from the South (150). Trainees from a cluster of southern and southeastern states, including Alabama, Arkansas, Florida, Georgia, Louisiana, Mississippi, Oklahoma, South Carolina, Tennessee, Texas, and West Virginia, are at significantly elevated injury risk during basic military training (151) and collectively they account for nearly 50% of total injury-related medical costs ($7,168,997 of $14,891,563 total) (150).

National Health and Nutrition Examination Survey (NHANES) data provide strong evidence that individuals living in southern states consistently have lower micronutrient intake and status compared with other regions in the United States. Although based on data collected more than three decades ago, analysis of NHANES III (1988 to 1994) showed that adults in the South consumed significantly lower amounts of calcium, magnesium, potassium, iron, and phosphorus, as well as vitamins A, C, K, and niacin, after adjusting for demographic and lifestyle factors compared with other regions (152). That these disparities have persisted is supported by longitudinal NHANES analyses spanning more than two decades, which further demonstrates that residents from southern states consistently exhibit lower blood concentrations of key micronutrients, including vitamins C, E, folate, and carotenoids, and poorer overall diet quality than those from other regions of the country, characterized by lower intake of fruits, vegetables, and whole grains (153). Many micronutrients play important structural and functional roles for bone (154) and many of those consistently deficient in southern states, including iron, vitamin K, and magnesium, are enzymatic cofactors responsible for bone metabolism, collagen synthesis, and collagen crosslinking (139). Low folate concentrations have also been associated with increased bone resorption markers in British Army basic military trainees, the implications of which, remain unclear (155). These population-level findings cannot be used to infer micronutrient deficiency in individual military trainees or to establish that regional differences in micronutrient status explain geographic variation in injury risk. Accordingly, rather than geographically targeted supplementation, these findings support an individualized approach in which nutritional status is screened and is assessed directly. Region of origin could serve as one screening variable alongside established individual-level risk factors, with nutritional assessment and intervention then guided by identified deficiencies or insufficiencies. Prospective studies incorporating individual-level measures of dietary intake, micronutrient status, fitness, body composition, training exposure, and injury outcomes are needed to determine whether nutritional disparities contribute to the elevated injury burden observed among trainees from southern states, and whether targeted nutritional interventions can reduce that risk.

### Other considerations

In 2018, the U.S. Army failed to meet its annual recruiting goal for the first time since 2005, underscoring growing concerns about the health and physical readiness of the U.S. recruiting pool. This shortfall prompted senior military leaders and policymakers to emphasize the importance of improving nutrition and physical activity during childhood and adolescence as foundational determinants of future military readiness (54, 150).

Evidence from athletic populations demonstrates that nutrition education can reduce bone stress injury risk when implemented comprehensively, and this is specifically pertinent to basic military trainees, as disordered eating patterns specifically in female trainees, are common (156) and a recent study demonstrated a high prevalence of recruits reporting intentional weight loss before starting basic military training, including through unhealthy mechanisms, such as skipping meals, using laxatives, and vomiting (157). In a multisite prospective study of female National Collegiate Athletic Association Division I endurance runners, a structured nutrition education intervention consisting of annual team-based presentations on energy availability and bone-supporting nutrients, combined with individualized sports registered dietitian counseling, reduced bone stress injury incidence at trabecular-rich sites from 0.18 to 0.10 events per person-year. Overall bone stress injury incidence also declined at one study site but not the other, attributed to differences in baseline injury rates, preexisting nutrition practices, and variability in intervention implementation. The authors noted that nutrition-related improvements in bone health may require multiple years to fully manifest, as restoring adequate energy availability and improving bone strength are gradual processes dependent on sustained behavioral change and cultural adoption (158). Thus, although comprehensive nutrition education represents a potentially valuable component of bone stress injury prevention, its effectiveness likely depends on consistent implementation, adequate resourcing, and sustained engagement. This represents an important implementation and evaluation gap in the military, where the effectiveness and consistency of existing nutrition initiatives remain incompletely characterized. A 2024 Government Accountability Office assessment concluded that the Department of Defense had not established department-wide strategic goals, performance goals, or performance metrics sufficient to fully evaluate the effectiveness of existing nutrition programs and initiatives (54).

Sleep restriction impairs endocrine homeostasis, compromising muscle regeneration, and uncoupling bone formation from resorption (159, 160). Robust evidence has consistently supported the relationship between shortened sleep duration and risk of bone stress injury in military personnel. For example, the Millennium Cohort Study, one of the most comprehensive studies of sleep in over 40,000 surveyed male (76%) and female (24%) service members, showed comorbidity was highest among short sleepers (< 6 h/night), and military personnel with documented MSKI reported sleeping fewer hours per night than their uninjured counterparts (161, 162). Among U.S. Army Special Forces personnel, of whom 95% were male, those who slept 4 h or less per night were 2.35 times more likely to suffer an MSKI than those who slept for 8 h or more per night (163). The Israeli Defense Forces credit a multi-component intervention, including an enforced sleep regimen of at least 6 h per night and a reduction in cumulative marching and running, with reducing stress fracture prevalence over the past 25 years, lowering fracture incidence in trainees by 62% compared with a 1983 historical cohort (30.8% to 11.6%) (164). Because these changes were implemented concurrently, the relative contribution of sleep cannot be separated from that of reduced training load, and the observed reduction should therefore be interpreted as the effect of the combined intervention rather than sleep alone. Importantly, existing data is almost exclusively in men, limiting generalizability to servicewomen who disproportionally suffer from endocrine dysfunction and bone stress injury (8, 165, 166) and sleep disorders (167) at a greater rate than their male counterparts. While the mechanisms by which insufficient sleep affects bone continue to be elucidated, proposed mechanisms include alterations in food restraint, meal timing, and energy intake (168, 169). While preliminary evidence in athletes suggests that improvements in sleep hygiene may enhance bone outcomes within relatively short timeframes (170), the efficacy of sleep extension or sleep quality improvements on bone strengthening during basic military training, and how sex may modify these relationships, remains to be seen.

### Limitations

Several limitations of this narrative review and the available evidence should be considered when interpreting the proposed framework. First, consistent with a narrative review approach, the literature search was not exhaustive or systematic, and studies were selected based on their relevance to the scope and objectives of the review. Consequently, selective inclusion cannot be excluded, and we performed no formal assessment of study quality or risk of bias. The military-specific literature is also drawn predominantly from U.S. service members and trainees, which may limit generalizability to other military populations with different training demands, occupational requirements, nutritional practices, and policies. Further, definitions, ascertainment methods, and diagnostic criteria for bone stress injuries and other MSKI vary across studies, limiting direct comparisons of injury incidence and intervention effects. Much of the available evidence relies on surrogate skeletal outcomes, including bone turnover markers, bone mineral density, and bone microarchitecture, rather than clinically diagnosed bone stress injuries or other MSKI. Relatively few randomized controlled trials have directly evaluated nutritional or other modifiable interventions for MSKI prevention in military populations. Accordingly, we incorporated non-military populations when relevant military-specific evidence was unavailable; however, differences in population characteristics, exposures, and outcomes limit the direct generalizability of these findings to service members. Finally, the quantity and nature of available evidence differ between men and women, which limits conclusions about potential sex-specific responses to several interventions. Collectively, these limitations should be considered when interpreting the proposed framework, which is intended to integrate the available evidence and identify opportunities for future research rather than establish definitive causal relationships or clinical practice recommendations.

### Conclusions and recommendations for future research

Nutritional bone stress injury prevention strategies in military settings have historically focused on a narrow set of isolated micronutrients, namely calcium and vitamin D, often without consistent surveillance, comprehensive dietary assessment, or integration with training load and recovery demands. The evidence reviewed here suggests that this siloed approach is insufficient and that a more comprehensive, longitudinal framework is needed.

Newly deposited bone requires at least one year to achieve full mechanical competence (115, 123), suggesting that nutritional interventions will be most effective when implemented well before training begins. However, to our knowledge, whether pre-enlistment nutritional intervention reduces bone stress injury risk in military recruits, and subsequent trainees, has not been directly established, though existing evidence is promising (124). Future research should therefore evaluate pre-enlistment and early-service nutritional programs, with recruitment as a potential entry point for standardized nutritional risk screening, dietary assessment, and targeted supplementation, particularly for recruits from high-incidence southern states where micronutrient insufficiency is regionally prevalent and disproportionate injury burden has been well-documented.

Early identification of vulnerability must be a foundational component of any prevention framework. Preexisting MSKI, nutritional deficiency, and poor physical preparation prior to basic military training have each been independently associated with injury risk and training attrition (4, 16, 178). The recently established Military Health and Nutrition Examination Study (MHANES) was designed to address the lack of comprehensive health and nutrition surveillance in active-duty soldiers by assessing dietary intake, body composition, biomarkers of nutritional status and health, injury prevalence, and physical performance in a representative Army sample, with the goal of informing evidence-based screening, intervention strategies, and policy decisions to improve Army health (171). Although MHANES is currently restricted to the U.S. Army, limiting generalizability to other services, its comprehensive integration of nutritional, physiological, injury, and performance outcomes provides an important foundation for identifying potentially modifiable nutritional correlates of injury and establishing military-specific health and nutrition benchmarks. Expansion to other services and repeated sampling cycles, as proposed by the investigators, could ultimately provide a military-wide surveillance framework for identifying emerging nutritional vulnerabilities, evaluating temporal trends, and informing targeted injury-prevention strategies.

Rapid escalation of training intensity and volume without adequate physical preparation is consistently reported antecedent to stress fracture, and this concern is increasingly important as contemporary basic military trainee populations trend towards lower baseline aerobic fitness and greater sedentary behavior than their predecessors (172, 173). Graduated progression in exercise volume and intensity represents a modifiable injury mitigation factor for which nutritional intervention alone cannot compensate (15). These findings suggest that incorporating a defined conditioning interval between MEPS processing and the start of formal training may allow for progressive physical adaptation alongside correction of nutritional deficiencies prior to the onset of training-related loading. Prospective evaluation, however, will be needed to determine whether such an interval reduces stress fracture incidence in military trainees.

Extending the pre-training period should not imply adoption of indiscriminate supplementation. Population-level strategies should prioritize access to nutritionally adequate diets that meet established requirements for energy, protein, calcium, vitamin D, iron, and other nutrients important to skeletal health, whereas higher-dose supplementation should be targeted to individuals with demonstrated inadequacy and accompanied by appropriate assessment and monitoring. This distinction is particularly important for iron. Although iron status can deteriorate during basic military training, particularly in women, randomized trials indicate that the greatest functional benefits of iron supplementation occur in trainees beginning training with iron deficiency or iron deficiency anemia, supporting identification and treatment of at-risk individuals rather than indiscriminate supplementation (174). Excessive iron intake can produce gastrointestinal adverse effects and, in susceptible individuals, contribute to iron overload; the tolerable upper intake level (UL) for healthy adults is 45 mg/day, although therapeutic doses above the UL may be clinically indicated for treatment of deficiency under appropriate supervision. Similar consideration applies to calcium and vitamin D, for which excessive supplemental intake can produce adverse effects, and established ULs should inform population-level interventions. Accordingly, implementation of pre-training nutritional interventions should consider baseline dietary intake and, where appropriate, biomarker status; supplement dose and duration; adherence and tolerability; potential nutrient interactions and established ULs; and the operational feasibility of screening, distribution, and longitudinal monitoring.

Accurate characterization of energy deficiency in military populations also remains a significant methodological challenge. Self-reported dietary intake has not been demonstrated to reliably predict stress fracture risk in male (72) or female (175) basic military trainees, and to date, no study has directly examined the effect of supplementary energy on bone health during basic military training. Given the high prevalence of intentional pre-enlistment weight loss including through disordered behaviors such as meal skipping and purging (157), the substantial heterogeneity between studies examining energy expenditure of women during basic military training specifically (176), and the documented high total energy expenditures exceeding ~4,700 and ~4,000 kcal·d^−1^ for female and male trainees, respectively (15, 177), improving objective methods for capturing energy deficit in field settings should remain a research priority.

Finally, nutritional strategies cannot be considered in isolation from behavioral and lifestyle factors that modulate bone adaptation to training. Insufficient sleep duration is independently associated with bone stress injury risk, partly through mechanisms involving disrupted endocrine homeostasis and altered eating behavior. Nutrition education has demonstrated efficacy in reducing bone stress injury incidence in collegiate endurance athletes when implemented comprehensively, consistently, and over multi-year timeframes. Military nutrition policy has evolved substantially, and targeted randomized military trials demonstrate that selected nutritional interventions can favorably affect injury incidence. However, evidence that implementation of broader nutrition policies has improved energy availability or nutrient adequacy, reduced calcium or vitamin D deficiency, decreased bone stress injury, or reduced injury-related attrition remains severely limited. Addressing these gaps will require coordinated efforts spanning nutrition science, sleep and behavioral medicine, and exercise physiology, alongside rigorous, adequately powered trials designed to evaluate combined interventions rather than isolated nutritional supplementation trials. Such an approach offers the most pragmatic way in which to reduce BSI incidence and preserve force readiness across the military training pipeline.
